# A call for action: understanding medicine’s role in climate change

**DOI:** 10.1007/s40037-019-00546-3

**Published:** 2019-11-21

**Authors:** Ciara Nuttall, Fatima Yaqub

**Affiliations:** Department of Medicine, Imperial College London, London, UK

We read with great interest the proposal to integrate climate change into medical school curricula [1]. The author focuses on teaching the impact the environment has on the health of the public; it is important to not miss the further opportunity of educating students on the impact healthcare systems such as the National Health Service (NHS) have on the environment.

The General Medical Council (GMC) code of ethics states we must ‘protect and promote the health of patients and the public’, so as doctors we implicitly have a responsibility to limit the damage we do to the health of the public. The NHS consumes massive amounts of energy and produces huge amounts of waste [2]. This is often unavoidable in order to maintain sterile conditions, however there is undeniably an excess. Students should be taught the impact the healthcare system as a whole has on the environment, the individual contributions that are made through our own choices and to close the loop, link this to the impact climate change has on global health.

On a minor scale, this may encourage students to be mindful of minimizing their own waste, for example returning unused equipment. Some may be empowered to conduct quality improvement projects during placements, focusing on environmental consumption and improving department efficiency. Importantly, students could learn to consider global health when making decisions as practising clinicians, for example prioritizing carbon neutrality or energy efficiency where possible.

Above influencing the activity of students as individuals, there is potential for wider-spread impact. As students become doctors and leaders within larger establishments, they have the power to induce greater changes and influence the direction the healthcare system takes. Pressure from the community is currently being focused on fuel and oil corporations, however public services also have a responsibility to become more sustainable. The NHS carbon reduction strategy was introduced 10 years ago [3], but since then sustainability has been put on a backburner in favour of more immediate concerns—in the NHS long-term plan, sustainability is only acknowledged in the appendix [4]. Prevention is key in the management of public health, and this is also true regarding global health. There must be top-down guidance combined with local empowerment in order to improve climate performance, which can be commenced by ensuring the medics of the future are fully informed.

## References

[1] Finkel ML (2019). A call for action: integrating climate change into the medical school curriculum. Perspect Med Educ.

[2] Pichler P, Jaccard I, Weisz U, Weisz H (2019). International comparison of health care carbon footprints. Environ Res Letters.

[3] Roberts I (2009). The NHS carbon reduction strategy. BMJ.

[4] Plan N (2019). NHS long term plan.

